# Assessment of Image-Guided Intratumoral Delivery of Immunotherapeutics in Patients With Cancer

**DOI:** 10.1001/jamanetworkopen.2020.7911

**Published:** 2020-07-29

**Authors:** Rahul A. Sheth, Ravi Murthy, David S. Hong, Sapna Patel, Michael J. Overman, Adi Diab, Patrick Hwu, Alda Tam

**Affiliations:** 1 Department of Interventional Radiology, University of Texas MD Anderson Cancer Center, Houston; 2 Department of Investigational Cancer Therapeutics, University of Texas MD Anderson Cancer Center, Houston; 3 Department of Melanoma Medical Oncology, University of Texas MD Anderson Cancer Center, Houston; 4 Department of Gastrointestinal Medical Oncology, University of Texas MD Anderson Cancer Center, Houston

## Abstract

**Importance:**

Direct intratumoral delivery of immunotherapies is a compelling approach to overcoming barriers to systemic immunotherapy efficacy. While the use of intratumoral delivery of immunotherapy drugs is increasing rapidly in both the investigational and standard of care domains, the feasibility and safety of these interventions, particularly for deeper lesions that require image-guidance, remain unknown.

**Objective:**

To address current knowledge gaps in image-guided techniques for intratumoral immunotherapy delivery and the safety of these interventions.

**Design, Setting, and Participants:**

This case series study was performed at a single tertiary cancer center over a 2-year period from January 2016 to January 2018. Patients were followed until January 2019. All patients who underwent image-guided intratumoral delivery of immunotherapy agents in the standard of care, off-label, or investigational setting during the study period were included. Data were analyzed from February 1 to June 1, 2019.

**Exposures:**

Image-guided biopsies and intratumoral injections of immunotherapies across several clinical trials as well as standard of care talimogene laherparepvec therapy.

**Main Outcomes and Measures:**

Technical success, defined as the delivery of the prescribed injectate volume in its entirety, for image-guided biopsy and injections and procedure-related adverse events.

**Results:**

A total of 85 patients (median [interquartile range] age, 61 [47-71] years; 42 [52%] men) underwent 498 encounters during the study period. These encounters comprised 327 image-guided intratumoral investigational agent injections in 67 patients in clinical trials, including 33 patients with melanoma (50%), 14 patients with sarcoma (21%), 3 patients with ovarian cancer (4.5%), 2 patients with breast cancer (3%), and 2 patients with colon cancer (3%). An additional 18 patients with melanoma underwent 113 image-guided talimogene laherparepvec injections. There were no adverse events reported related to the technical component of the procedure, specifically needle insertion or biopsy. Serious adverse events (Common Terminology Criteria for Adverse Events score ≥3), including dyspnea and severe flu-like symptoms developing within 24 hours of the injection and requiring hospitalization, occurred after 3 of 327 investigational agent injections (2%) and 4 of 113 talimogene laherparepvec injections (4%).

**Conclusions and Relevance:**

The findings of this case series study suggest that intratumoral injections of immunotherapies were feasible across a range of histological conditions and target organs. Immediate postdelivery anticipated adverse events occurred in a small number of instances. Performing physicians should have the necessary safeguards in place to respond as needed. Optimal methods for intratumoral drug delivery remain unresolved, and efforts to standardize drug delivery techniques are required.

## Key Points

**Question:**

What is the feasibility and safety of image-guided intratumoral delivery of immunotherapies in patients with advanced solid organ malignancies?

**Findings:**

In this case series study that included 85 patients, there were no adverse events related to the technical component of the procedure, specifically needle insertion or biopsy. Major adverse events due to immune-related systemic toxic effects occurred in 2% of investigational agents and 4% of standard-of-care procedures (4%).

**Meaning:**

Intratumoral delivery of immunotherapies is technically feasible with low major complication rates.

## Teaser

This case series study examines the feasibility and safety of intratumoral delivery of immunotherapy among patients undergoing cancer care in clinical trials.

## Introduction

The conventional method for delivery of chemotherapy via intravenous administration allows for the distribution of the treatment drug throughout the body, but known challenges limit its effectiveness. Drug penetration into the tumor tissue can be inadequate owing to inherent barriers imposed by the tumor microenvironment.^1,2^ Additionally, indiscriminate exposure of the nontumorous compartment often adversely influences the agents’ effectiveness or results in prohibitive toxic effects. Local intratumoral injection of anticancer therapies is a logical solution to overcoming these barriers to drug delivery.^3^ With the recent advancements in immune-based cancer therapies, there has been a resurgence of interest in the delivery of therapeutic agents directly into tumors, either as primary therapy or as an adjunct agent in combination with systemic immunotherapy.^4^ By successfully delivering a high concentration of immunostimulatory agents into a tumor site, local intratumoral drug delivery has the potential to drive sustained, systemic immune responses.^5^

The past 5 years have witnessed a tremendous proliferation of intratumoral immunotherapies. Types of agents are varied and range from cytokines and monoclonal antibodies to oncolytic viruses, cell-based therapies, and nanoparticles.^6^ There are more than 20 ongoing clinical trials for intratumoral injection of oncolytic viruses alone, and in 2015, the US Food and Drug Administration approved the first oncolytic virus, talimogene laherparepvec (TVEC), as immunotherapy for the treatment of patients with metastatic melanoma that cannot be surgically removed.^7^

To our knowledge, most studies investigating intratumoral injections have included patients with melanoma who have had injections to palpable subcutaneous lesions. As intratumoral injections expand from patients with melanoma to patients with other solid organ malignant tumors and lymphomas, there is increasing interest to perform injections to deeper targets using image guidance. However, little is known about the safety and feasibility of performing image-guided intratumoral injections. As intratumoral immunotherapy clinical protocols typically require repeated drug administrations, there are legitimate concerns regarding the risks of bleeding and organ injury due to frequent, repeated needle punctures. Furthermore, given the increased vascularity of visceral organs, such as the liver, compared with the dermis, there are likewise legitimate concerns about intravasation of the intratumoral drug resulting in systemic toxic effects.

In this case series, we present our single-institution experience with image-guided intratumoral injections in the investigational, off-label, or standard-of-care settings in patients with solid tumors. We present our institution’s experience with performing image-guided intratumoral injections in a cohort of patients that spans a range of malignant tumors, injection sites, and immunotherapeutics. As this study represents the first characterization of such a cohort to our knowledge, the purpose of this study is to address current knowledge gaps in image-guided techniques and the safety of these interventions.

## Methods

The MD Anderson Cancer Center review board approval was obtained for this single-institution, retrospective study. Waiver for informed consent was granted by the institutional review board because this study satisfied the criteria encoded in the Code of Federal Regulations Protection of Human Subjects 45CFR46. This report follows the reporting guideline for case series.^8^

### Study Population

An institutional radiology database was queried for patients who underwent image-guided intratumoral delivery of immunotherapy agents over a 2-year period from January 2016 to January 2018.^9,10,11,12,13,14,15,16,17,18,19^ In addition, 4 patients who underwent image-guided intratumoral drug delivery in a clinical trial assessing PV-10 chemoablation of liver cancer^20^ from May to October 2018 were included in this study; given the study drug’s iodine content, this study provided a unique opportunity to visualize intratumoral drug distribution with intraprocedural computed tomography imaging. Patients who underwent intratumoral injection without image guidance (ie, by palpation alone) were excluded. Patients were followed by health record review until January 30, 2019. Evidence of adverse events recorded in the postanesthesia care unit as well as emergency department visits within 24 hours of the procedure were noted. Complications within 24 hours of the injection or trial-mandated biopsy were evaluated using the National Cancer Institute Common Terminology Criteria for Adverse Events scoring system, version 5.0.

### Intratumoral Injections

The selection of tumor lesions for intratumoral drug delivery or biopsy was made collaboratively by the patient’s primary oncologist and an interventional radiologist. The appropriate modality for image guidance during the injection procedure was determined by the interventional radiologist. Interventions on subcutaneous lesions were performed with local anesthesia (1% lidocaine) only, while those performed on deeper lesions or lesions within visceral organs were performed with conscious sedation (fentanyl/versed) or monitored anesthesia care.

All intratumoral injection procedures were performed by interventional radiologists with 2 to 20 years of experience in image-guided interventions. The technique for intratumoral drug delivery was performed per clinical trial protocol when specified or by the operator’s best judgement when not specified. Technical success for intratumoral injection procedures was defined as the delivery of the prescribed injectate volume in its entirety. Technical success for clinical trial protocol-mandated biopsies was the acquisition of the requisite tissue samples.

### Statistical Analysis

Quantitative, nonnormally distributed data were summarized by their medians and interquartile ranges (IQRs) or ranges. Categorical data were analyzed as percentages. Data were analyzed using R statistical software version 3.5.1 (R Foundation for Statistical Computing) from February 1 to June 1, 2019.

## Results

**Figure 1.**
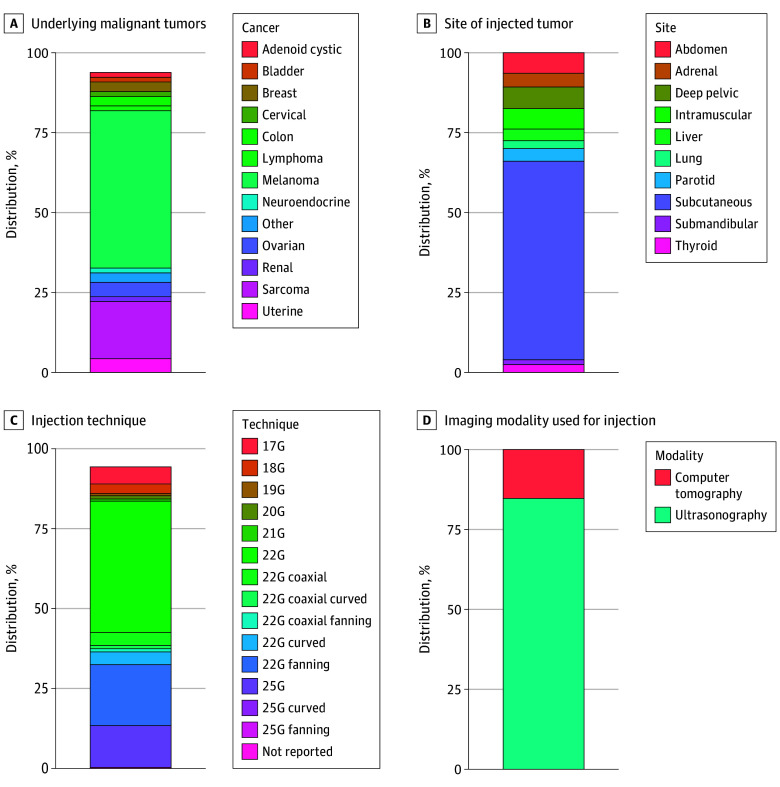
Distribution of Cancer Histological Conditions

A total of 85 patients (median [IQR] age, 61 [47-71] years, 42 [52%] men) underwent 498 encounters during the study period. This included 67 patients in clinical trials who underwent 385 encounters, with 327 image-guided intratumoral investigational agent injections (Figure 1) and 192 image-guided biopsies across 12 clinical trials (Table 1).^9,10,11,12,13,14,15,16,17,18,19,20^ Malignant tumors included melanoma (33 patients [50%]), sarcoma (14 patients [21%]), ovarian cancer (3 patients [5%]), breast cancer (2 patients [3%]), colon cancer (2 patients [3%]), and other cancer (13 patients [19%]). Additionally, 18 patients (9 patients with cutaneous melanoma as standard-of-care, 9 patients with uveal melanoma as off-label use) underwent 113 image-guided intratumoral injections of TVEC. The median (range) number of encounters per patient was 6 (1-20) in the investigational setting and 5 (1-17) for TVEC. Subcutaneous lesions that required image guidance for intratumoral injection included 203 of 327 tumors (62%) in patients in clinical trials and 18 tumors (100%) in patients who received TVEC. Among 327 total image-guided injections, visceral lesions in deeper locations and solid organs were also injected pelvically (22 injections [7%]), abdominally (21 injections [6%]), intramuscularly (21 injections [6%]), adrenally (14 injections [4%]), in the liver (12 injections [4%]), and in the lung (8 injections [2%]). Among patients in clinical trials, the median (range) target lesion tumor volume was 6.4 (0.1-984) cm^3^, and the median (range) target lesion length was 3.3 (0.9-12.8) cm; the median (range) injected volume was 2.0 (0.5-4.0) mL. Among patients who received TVEC, the median (range) target lesion tumor volume was 3.5 (0.2-250) cm^3^, and the median (range) target lesion length was 2.4 (0.8-8.0) cm; the median (range) injected volume was 0.5 (0.3-4.0) mL.

**Table 1.** Summary of the Clinical Trials, Their Investigational Immunotherapy Agents, and Instructions for Intratumoral Delivery Technique Based on the Information Provided by the Study Protocol

Trial	NCT No.	Intratumoral Agent	Phase	Specified injection technique
Safety study of intratumoral injection of clostridium novyi-NT spores to treat patients with solid tumors that have not responded to standard therapies^9^	NCT01924689	Clostridium novyi-NT spores	I	22-24G needles; injection may be redirected to 4 distinct sites in the tumor
Phase 1b safety study of CMB305 in patients with locally advanced, relapsed, or metastatic cancer expressing NY-ESO-1^10^	NCT02387125	G100	I	Inject slowly; needle may be repositioned
A study to assess the safety and efficacy of intratumoral IMO-2125 in combination with ipilimumab in patients with metastatic melanoma (ILLUMINATE-204)^11^	NCT02644967	IMO-2125	I/II	25G needle recommended, but choice at IR’s discretion; coaxial with curved needle and fanning technique specified
APX005M in combination with systemic pembrolizumab in patients with metastatic melanoma^12^	NCT02706353	APX005M	I/II	None specified
A study of MEDI9197 in subjects with solid tumors or CTCL and in combination with durvalumab and/or palliative radiation in subjects with solid tumors^13^	NCT02556463	MEDI9197	I	22G or 25G needle; inject under Doppler ultrasonographic guidance
Safety and efficacy of MIW815 (ADU-S100) +/− ipilimumab in patients with advanced/metastatic solid tumors or lymphomas^14^	NCT02675439	MIW815 (ADU-S100)	I	None specified
Safety, tolerability, PK, dosimetry, MTD and preliminary efficacy of intra-lesionally injected AvidinOX, followed by IV escalating doses of [177Lu]DOTA-biotin in pts with injectable solid tumors or lymphomas^15^	NCT03188328	AvidinOX	I	Multihole needle should be used (no specifics provided)
A study of ABBV-927 and ABBV-181, an immunotherapy, in subjects with advanced solid tumors^16^	NCT02988960	ABBV-927	I	Needle size, injection rate, and duration of injection at IR’s discretion
Study of the safety and efficacy of MIW815 with PDR001 to patients with advanced/metastatic solid tumors or lymphomas^17^	NCT03172936	MIW815 (ADU-S100)	I	None specified
A study of intratumoral IMO-2125 in patients with refractory solid tumors (ILLUMINATE-101)^18^	NCT03052205	IMO-2125 (single agent)	I	25G needle recommended but choice at IR’s discretion; fanning technique specified
A Study of Toca 511, a retroviral replicating vector, combined with Toca FC in patients with solid tumors or lymphoma (Toca 6)^19^	NCT02576665	Vocimagene amiretrorepvec (Toca 511)	I	No titanium needles; inject slowly, consider fanning method over 5-10 min
A study to assess PV-10 chemoablation of cancer of the liver^20^	NCT00986661	PV-10 (rose bengal disodium)	I	End-hole or multipronged needles may be used

Abbreviations: IR, interventional radiologist; PK, pharmacokinetics; MTD, maximum tolerated dose; IV, intravenous; pts, patients.

**Figure 2.**
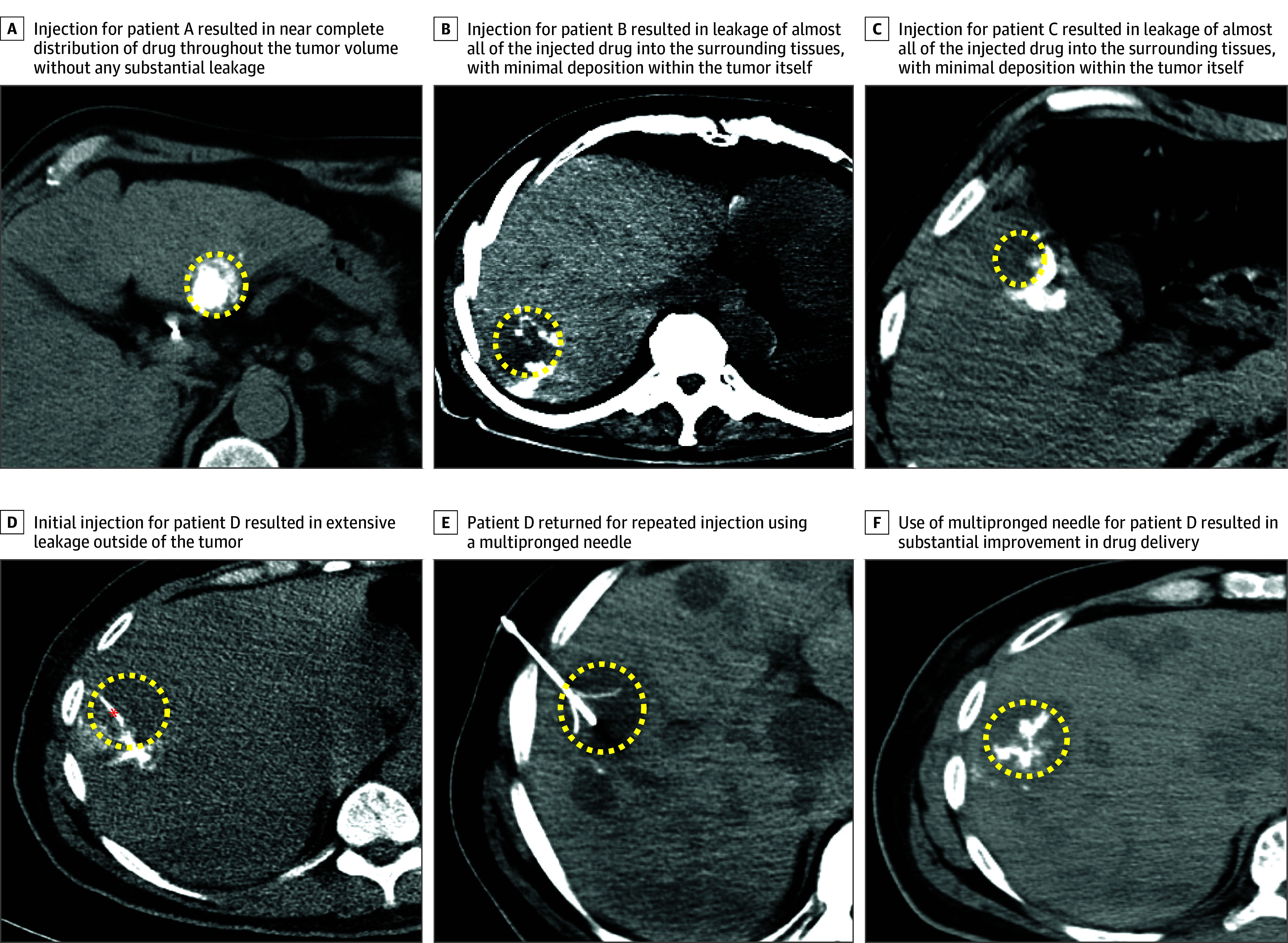
Spectrum of Intratumoral Drug Distribution and Influence of Injection Technique Intra-procedural non-contrast enhanced axial computed tomography images from 4 patients (A-F) demonstrate the range of intratumoral distribution outcomes. Target tumors are outlined in dotted yellow ellipses. Injection for patient A resulted in near complete distribution of drug throughout the tumor volume without any substantial leakage. Injections for patients B and C resulted in leakage of almost all of the injected drug into the surrounding tissues, with minimal deposition within the tumor itself. For patient D, initial injection using a 21G end-hole needle (red asterisk) resulted in extensive leakage outside of the tumor (D). The patient returned for repeat injection using a multi-pronged needle (E), which resulted in substantial improvement in intratumoral drug delivery (F).

A variety of injection methods were used to optimize distribution within the lesion when using a single end-hole beveled needle (Figure 1). As most of immunotherapeutic agents in this study could not be directly visualized by conventional noninvasive imaging techniques, we were unable to directly assess the distribution and retention of the drug within the tumor after injection. However, as 1 investigational agent contained iodine,^20^ it could be detected by computed tomography. A tracer and fanning method was used to optimize distribution within the lesion using a single end-hole beveled needle for patients in this trial. Injections in this trial revealed a wide spectrum of intratumoral delivery outcomes (Figure 2). While some injections resulted in near-complete filling of the tumor volume with the drug, other injections led to leakage of the drug into the surrounding parenchyma, with minimal drug retention within the target tumor. In 1 patient, after poor intratumoral delivery using a tracer and fanning method with a single end-hole needle, a second injection was performed using a multipronged injection needle (Quadrafuse; Rex Medical); this resulted in a substantial improvement in intratumoral drug delivery.

There were no adverse events reported associated with the technical component of the procedure (ie, needle insertion or biopsy). However, serious adverse events (Common Terminology Criteria for Adverse Events score ≥3), including dyspnea and severe flu-like symptoms developing within 24 hours of the injection and requiring hospitalization, occurred after 5 of 327 investigational agent injections (2%) and 4 of 113 TVEC injections (4%) (Table 2). Seven patients treated with investigational agents developed several adverse reactions immediately after intratumoral drug delivery resembling cytokine release syndrome. For example, 1 patient developed an immediate adverse reaction in the procedure room within minutes of receiving the intratumoral injection. Notably, the patient had received the same investigational agent to the same tumor site 4 times previously without an adverse reaction. The patient became tachycardic, hypotensive (ie, systolic blood pressure in the 70s mm Hg), and diaphoretic. The patient was resuscitated with intravenous fluids, supplemental oxygen, and intravenous diphenhydramine and was admitted to the hospital. The patient was treated conservatively and discharged in stable condition 24 hours after the procedure.

**Table 2.** Summary of AEs Within 24 Hours of Intratumoral Injections^a^

Injection site	Adverse event	De novo IRAE	First injection	Cancer histology
TVEC				
Pelvic lymph node	Fever, vomiting, and headache requiring presentation to emergency center; managed with IV fluids and antibiotics	Yes	Yes; subsequent 4 injections without AE	Cutaneous melanoma
Pelvic lymph node	Malaise and fever requiring presentation to emergency center and hospitalization	Yes	No; 1 prior injection without AE	Cutaneous melanoma
Chest wall lesion	Tachypnea and tachycardia requiring presentation to emergency center and hospitalization	No, existing chronic lung disease	No; 1 prior and 4 subsequent injections without AE	Uveal melanoma
Calf subcutaneous lesion	Asymptomatic hypertension (systolic 180s mm Hg) requiring IV antihypertensives and monitoring	Yes	No; 3 prior and 4 subsequent injections without AE	Uveal melanoma
Investigational agents				
Axillary lesion	Dyspnea, rigors, and tachycardia requiring presentation to emergency center and hospitalization	Yes	Yes; 3 subsequent injections without AE	Cutaneous melanoma
Thigh subcutaneous lesion	Hives and rigors, managed with antihistamines and overnight observation	Yes	No; 5 prior injections without AE	Melanoma
Liver lesion	Tachycardia and chills requiring hospitalization	Yes	No; 1 prior and one subsequent injection without AE	Colon cancer
Thigh subcutaneous lesion	Tachycardia and hypertension followed by symptomatic hypotension (systolic blood pressure in the 70s mm Hg); managed with fluids, antihistamines, and hospitalization	Yes	No; 4 prior injections without AE	Melanoma
Head and neck lesion	Hypotension (systolic blood pressure in the 60s mm Hg) and rigors, requiring admission to intensive care unit and managed with steroids for adrenal insufficiency	No, history of adrenal insufficiency from prior checkpoint inhibitor therapy	Yes; 5 subsequent injections without AE	Melanoma

Abbreviations: AE, adverse event; IRAE, immune-related adverse event; IV, intravenous; TVEC, talimogene laherparepvec.

^a^ Adverse events were defined as those with Common Terminology Criteria for Adverse Events score of 3 or greater.

One patient in an investigational clinical trial presented for a 24-hour postinjection biopsy procedure but was found to be symptomatically hypotensive with systolic blood pressures in the 60s mm Hg and was also noted to have rigors. The patient’s prior history of immune checkpoint inhibitor therapy related-adrenal insufficiency lowered their threshold for adverse events, and they were successfully treated with fluids and corticosteroids and discharged after a brief admission in the intensive care unit.

Minor adverse events (Common Terminology Criteria for Adverse Events score <3), including allergic reactions that did not require an escalation of care, occurred in 11 of 327 (3%) clinical trial injections and 1 of 113 (1%) TVEC injections.

## Discussion

The findings of this case series suggest that intratumoral injections of immunotherapies are feasible across a range of histological conditions and target organs. While the adverse event rate was low, performing physicians need to be aware and equipped to handle these types of systemic immune-related reactions in addition to typical postprocedure complications.

The optimal method for drug delivery that maximizes intratumoral drug distribution and retention remains unresolved. As with any invasive intervention, outcomes after intratumoral drug delivery depend on procedural technique. This is corroborated by the substantial difference in intratumoral drug delivery outcome observed when the same tumor in the same patient was injected with 2 different injection techniques. However, detailed and standardized guidelines for intratumoral injection methods are lacking. The negative ramifications of imperfect procedural technique are 2-fold. Limited delivery of the immunotherapy drug to its target site may result in diminished efficacy, but a more immediate challenge is the potential for systemic exposure to high doses of these very potent drugs. Efforts to standardize drug delivery techniques may be required.

### Limitations

This study has several limitations. Given the diversity of intratumoral immunotherapies in development, each with its own safety profile, it is challenging to extrapolate the outcomes of this study broadly across the spectrum of intratumoral therapies. For example, given the myriad mechanisms of action, the degree to which off-target deposition can lead to systemic toxic effects is likely highly variable from one therapy to another. Furthermore, while this study’s low procedural complication rate represents an important finding, it precludes further analysis to identify technical factors, such as lesion location, lesion size, or injection technique, that may be associated with greater risk for complications.

## Conclusions

Our findings in approximately 500 intratumoral injections during a 2-year period demonstrates that multiple image-guided intratumoral injections, including tumors in deep and visceral organ locations, are feasible for patients with advanced solid tumors. Incorporating standardized, evidence-based instructions for drug delivery technique into investigational and standard-of-care protocols will be essential to optimizing the efficacy of intratumoral therapy.

## References

[1] Sheth RA, Hesketh R, Kong DS, Wicky S, Oklu R. Barriers to drug delivery in interventional oncology. J Vasc Interv Radiol. 2013;24(8):1201-1207. doi:10.1016/j.jvir.2013.03.034 23735316

[2] Minchinton AI, Tannock IF. Drug penetration in solid tumours. Nat Rev Cancer. 2006;6(8):583-592. doi:10.1038/nrc1893 16862189

[3] Bilusic M, Gulley JL. Editorial: local immunotherapy: a way to convert tumors from “cold” to “hot”. J Natl Cancer Inst. 2017;109(12):1-2. doi:10.1093/jnci/djx132 30053078 PMC6279267

[4] Marabelle A, Andtbacka R, Harrington K, . Starting the fight in the tumor: expert recommendations for the development of human intratumoral immunotherapy (HIT-IT). Ann Oncol. 2018;29(11):2163-2174. doi:10.1093/annonc/mdy423 30295695 PMC6290929

[5] Ribas A, Dummer R, Puzanov I, . Oncolytic virotherapy promotes intratumoral T cell infiltration and improves anti-PD-1 immunotherapy. Cell. 2017;170(6):1109-1119.e10. doi:10.1016/j.cell.2017.08.027 28886381 PMC8034392

[6] Murthy V, Minehart J, Sterman DH. Local immunotherapy of cancer: innovative approaches to harnessing tumor-specific immune responses. J Natl Cancer Inst. 2017;109(12):1-12. doi:10.1093/jnci/djx097 29546344

[7] Andtbacka RHI, Ross M, Puzanov I, . Patterns of clinical response with talimogene laherparepvec (T-VEC) in patients with melanoma treated in the OPTiM phase III clinical trial. Ann Surg Oncol. 2016;23(13):4169-4177. doi:10.1245/s10434-016-5286-0 27342831 PMC5090012

[8] Kempen JH. Appropriate use and reporting of uncontrolled case series in the medical literature. Am J Ophthalmol. 2011;151(1):7-10.e1. doi:10.1016/j.ajo.2010.08.047 21163373 PMC3052978

[9] ClinicalTrials.gov. Safety study of intratumoral injection of Clostridium novyi-NT spores to treat patients with solid tumors that have not responded to standard therapies. Accessed January 31, 2020. https://clinicaltrials.gov/ct2/show/NCT01924689

[10] ClinicalTrials.gov. Phase 1b safety study of CMB305 in patients with locally advanced, relapsed, or metastatic cancer expressing NY-ESO-1. Accessed January 31, 2020. https://clinicaltrials.gov/ct2/show/NCT02387125 10.1080/2162402X.2020.1847846 PMC7714520 33312760

[11] ClinicalTrials.gov. A study to assess the safety and efficacy of intratumoral IMO-2125 in combination with ipilimumab or pembrolizumab in patients with metastatic melanoma (ILLUMINATE-204). Accessed January 31, 2020. https://clinicaltrials.gov/ct2/show/NCT02644967

[12] ClinicalTrials.gov. APX005M in combination with systemic pembrolizumab in patients with metastatic melanoma. Accessed January 31, 2020. https://clinicaltrials.gov/ct2/show/NCT02706353

[13] ClinicalTrials.gov. A study of MEDI9197 in subjects with solid tumors or CTCL and in combination with durvalumab and/or palliative radiation in subjects with solid tumors. Accessed January 31, 2020. https://clinicaltrials.gov/ct2/show/NCT02556463

[14] ClinicalTrials.gov. Safety and efficacy of MIW815 (ADU-S100) +/- ipilimumab in patients with advanced/metastatic solid tumors or lymphomas. Accessed January 31, 2020. https://clinicaltrials.gov/ct2/show/NCT02675439

[15] ClinicalTrials.gov. Safety, tolerability, PK, dosimetry, MTD and preliminary efficacy of intra-lesionally injected AvidinOX, followed by IV escalating doses of [177Lu]DOTA-biotin in pts with injectable solid tumors or lymphomas. Accessed January 31, 2020. https://clinicaltrials.gov/ct2/show/NCT03188328

[16] ClinicalTrials.gov. A study of ABBV-927 and ABBV-181, an immunotherapy, in participants with advanced solid tumors. Accessed January 31, 2020. https://clinicaltrials.gov/ct2/show/NCT02988960

[17] ClinicalTrials.gov. Study of the safety and efficacy of MIW815 with PDR001 to patients with advanced/metastatic solid tumors or lymphomas. Accessed January 31, 2020. https://clinicaltrials.gov/ct2/show/NCT03172936

[18] ClinicalTrials.gov. A study of intratumoral IMO-2125 in patients with refractory solid tumors (ILLUMINATE-101). Accessed January 31, 2020. https://clinicaltrials.gov/ct2/show/NCT03052205

[19] ClinicalTrials.gov. A study of Toca 511, a retroviral replicating vector, combined with Toca FC in patients with solid tumors or lymphoma (Toca 6). Accessed January 31, 2020. https://clinicaltrials.gov/ct2/show/NCT02576665

[20] ClinicalTrials.gov. A study to assess PV-10 chemoablation of cancer of the liver. Accessed January 31, 2020. https://clinicaltrials.gov/ct2/show/NCT00986661

